# JAK-STAT1 Signaling Pathway Is an Early Response to *Helicobacter pylori* Infection and Contributes to Immune Escape and Gastric Carcinogenesis

**DOI:** 10.3390/ijms23084147

**Published:** 2022-04-08

**Authors:** Xue Li, Kaifeng Pan, Michael Vieth, Markus Gerhard, Wenqing Li, Raquel Mejías-Luque

**Affiliations:** 1 Key Laboratory of Carcinogenesis and Translational Research (Ministry of Education/Beijing), Department of Cancer Epidemiology, Peking University Cancer Hospital and Institute, Beijing 100142, China; xue.li@bjmu.edu.cn (X.L.); pankaifeng2002@yahoo.com (K.P.); 2 PYLOTUM Key Joint Laboratory for Upper GI Cancer, Peking University Cancer Hospital and Institute, Beijing 100142, China; michael.vieth@uni-bayreuth.de (M.V.); markus.gerhard@tum.de (M.G.); 3 Institute for Medical Microbiology, Immunology and Hygiene, School of Medicine, Technical University of Munich (TUM), 81675 Munich, Germany; 4 Institute of Pathology, Friedrich-Alexander University Erlangen-Nuremberg, Klinikum Bayreuth, 95445 Bayreuth, Germany

**Keywords:** gastric cancer, STAT1, PD-L1

## Abstract

*Helicobacter pylori* infection induces a number of pro-inflammatory signaling pathways contributing to gastric inflammation and carcinogenesis and has been identified as a major risk factor for the development of gastric cancer (GC). Janus kinase-signal transducer and activator of transcription (JAK-STAT) signaling mediates immune regulatory processes, including tumor-driven immune escape. Programmed death ligand 1 (PD-L1) expressed on gastric epithelium can suppress the immune system by shutting down T cell effector function. In a human cohort of subjects with gastric lesions and GC analyzed by proteomics, STAT1 increased along the cascade of progression of precancerous gastric lesions to GC and was further associated with a poor prognosis of GC (Hazard Ratio (95% confidence interval): 2.34 (1.04–5.30)). We observed that STAT1 was activated in human *H. pylori*-positive gastritis, while in GC, STAT1, and its target gene, PD-L1, were significantly elevated. To confirm the dependency of *H. pylori*, we infected gastric epithelial cells in vitro and observed strong activation of STAT1 and upregulation of PD-L1, which depended on cytokines produced by immune cells. To investigate the correlation of immune infiltration with STAT1 activation and PD-L1 expression, we employed a mouse model of *H. pylori*-induced gastric lesions in an Rnf43-deficient background. Here, phosphorylated STAT1 and PD-L1 were correlated with immune infiltration and proliferation. STAT1 and PD-L1 were upregulated in gastric tumor tissues compared with normal tissues and were associated with immune infiltration and poor prognosis based on the TCGA-STAD database. *H. pylori*-induced activation of STAT1 and PD-L1 expression may prevent immune surveillance in the gastric mucosa, allowing premalignant lesions to progress to gastric cancer.

## 1. Introduction

Gastric cancer (GC), especially the intestinal type, is a highly heterogeneous disease preceded by a prolonged, multistage precancerous process, including superficial gastritis (SG), chronic atrophic gastritis (CAG), intestinal metaplasia (IM), and dysplasia [1,2,3]. *Helicobacter pylori* infection-induced chronic inflammation is the most recognized risk factor for the progression of gastric lesions and the development of GC [4]. Although the correlation between GC and *H. pylori* infection has been extensively confirmed [5], the molecular mechanisms underlying the pathogenesis of GC remain to be fully defined.

Chronic inflammation and persistent bacterial infection lead to the progression of gastritis to IM and GC. This process is supported by the activation of a number of pro-inflammatory signaling pathways by *H. pylori,* which are important for the recruitment of immune cells to the gastric mucosa. Among those, interferon gamma (IFN-γ)-producing T cells are considered responsible for much of the pathology inflicted by *H. pylori* infection [6]. IFN-γ produced by infiltrating lymphocytes activates STAT1 through the Janus kinase (JAK)-mediated phosphorylation of tyrosine (Y) 701. The IFN-γ receptor employs JAK1 and JAK2 to phosphorylate exclusively STAT1, causing its homodimerization. Phosphorylated STAT1 (p-STAT1) homodimers can translocate into the nucleus, where they bind to DNA, promote transcription of target genes, and induce the expression of proteins that affect basic cell activities and immune responses [7]. While some studies showed that STAT1 is induced in epithelial cells upon *H. pylori* infection [8,9], other studies claimed a reduction of the activation of STAT1 upon infection with *H. pylori* strains proficient in a type IV secretion system and Cytotoxin associated gene A (CagA) [10,11,12]. Therefore, further insight into the possible regulation of STAT1 activation by *H. pylori* is necessary. In addition, considering the involvement of STAT1 in anti-tumor immune responses, the identification of the molecular mechanisms underlying the contribution of *H. pylori*-mediated STAT1 regulation to tumor progression and immune escape is important for designing novel therapeutic interventions for GC patients. In this context, previous studies have shown that STAT1 induces programmed death-ligand 1 (PD-L1) on epithelial cells [13,14]. PD-L1 is an important regulator of CD8 T-cell functionality, and its expression in the stomach is associated with T cell inhibition [15,16,17]. However, no data on the possible regulation of PD-L1 expression by STAT1 in the context of *H. pylori*-driven gastric carcinogenesis has been reported.

We hypothesize that the STAT1 signaling pathway is an early response to *H. pylori* infection and, when accompanied by PD-L1 expression, may protect the gastric epithelium from cytotoxic CD8+ T-cell responses, and allow premalignant lesions to progress to GC.

## 2. Results

### 2.1. H. pylori Infection Induces STAT1 Activation and PD-L1 Expression on Gastric Epithelial Cells in an Immune-Cell Dependent Manner

**Figure 1:**
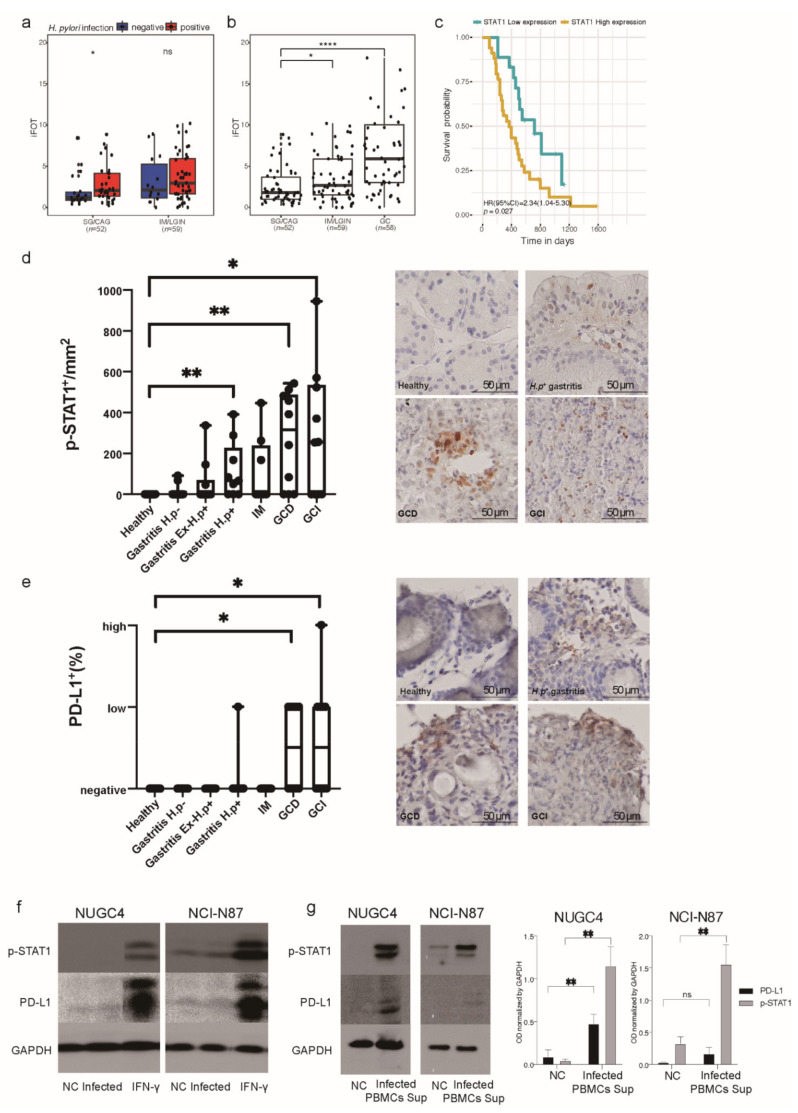
STAT1 in the human gastric tissue proteomic cohort and immunohistochemistry and Western blot analysis of p-STAT1 and PD-L1 in gastric biopsies and gastric epithelial cells after *H. pylori* infection. (**a**) The expression of STAT1 in *H. pylori*-positive subjects and negative subjects, stratified by pathology. (**b**) STAT1 expression in gastritis (SG/CAG), advanced gastric lesions (IM/LGIN) and GC. (**c**) Kaplan–Meier curves of STAT1 in prognosis analysis of GC. (**d**,**e**) Immunohistochemistry staining of p-STAT1 and PD-L1 in human gastric biopsies (20× magnification). Healthy (*n* = 10), Gastritis *H.p*− (*n* = 10), Gastritis EX-*H.p*+ (*n* = 10), Gastritis *H.p*+ (*n* = 9), IM (*n* = 8), GCD (*n* = 10), GCI (*n* = 10). Wilcoxon rank-sum test, ns, non-significant, * *p* < 0.05, ** *p* < 0.01, **** *p* < 0.0001. PD-L1 staining evaluation: <1%, negative; 1–49% low; ≥50% high. (**f**) Western blot analysis of p-STAT1 and PD-L1 in NUGC4 and NCI-N87 negative controls and after 24 h *H. pylori* infection. IFN-γ (10 ng/mL) cells stimulated for 24 h were used as positive control. (**g**) Western blot analysis of p-STAT1 and PD-L1 in NUGC4 and NCI-N87 incubated 24 h with supernatants from non-infected or *H. pylori-infected* PBMCs for 24 h. GAPDH was used as loading control. Graphs show the mean ± SD of three independent experiments. Student’s *t* test, ns, non-significant, * *p* < 0.05, ** *p* < 0.01. CAG, chronic atrophic gastritis; CI, confidence interval; EX-*H.p*+, previously *H.p* positive; GC, gastric cancer; GCD, diffuse-type gastric cancer; GCI, intestinal-type gastric cancer; *H. p*, *Helicobacter pylori*; HR, hazard ratio; iFOT, intensity-based fraction of total; IM, intestinal metaplasia; LGIN, low-grade intraepithelial neoplasia; NC, negative control; PBMCs, peripheral blood mononuclear cells; SG, superficial gastritis; Sup, supernatant.

We previously established a human gastric tissue proteomic cohort of 169 subjects, including 33 with SG, 19 with CAG, 56 with IM, 3 with low-grade intraepithelial neoplasia (LGIN), and 58 with GC, to explore molecular signatures associated with the progression of gastric lesions and risk of early GC [18]. In this cohort, we observed that STAT1 expression was significantly upregulated in *H. pylori*-positive gastritis compared to *H. pylori*-negative gastritis (Figure 1a). Interestingly, the expression of STAT1 was found to increase along the cascade of progression of gastric lesions to GC (Figure 1b). We next analyzed tumor prognosis and identified STAT1 to be independently associated with poor prognosis of GC with a hazard ratio (HR) (95% confidence interval [CI]) of 2.34 (1.04–5.30) after adjusting for age, sex, TNM stage, and Lauren type (Figure 1c). We hypothesized that STAT1 could represent an early response marker to *H. pylori* infection and favor the development of precancerous gastric lesions progressing to GC.

To confirm STAT1 activation in response to *H. pylori* infection and during the course of gastric carcinogenesis, we stained p-STAT1 in human stomach biopsies. STAT1 was found to be activated in epithelial cells especially in *H. pylori* positive gastritis and was significantly upregulated in both diffuse type and intestinal type GC (Figure 1d).

We next explored PD-L1 expression in the same tissue samples, as it was previously shown that STAT1 regulates its expression in other tumor types. PD-L1 was also significantly upregulated in GC compared to healthy mucosa (Figure 1e). These observations indicate that STAT1 activation may be an early response to *H. pylori* infection, while PD-L1 expression occurs only at later stages of progression to GC.

To explore the role of gastric epithelial cells and immune cells in the activation of STAT1 and the induction of PD-L1 expression, we first infected gastric cancer cell lines NUGC4 and NCI-N87 with the *H. pylori* G27 strain and detected p-STAT1 and PD-L1 expression by western blot. Compared to negative controls, we did not observe either activation or upregulation of STAT1 or expression of PD-L1 in *H. pylori*-infected GC cells (Figure 1f). We then isolated human peripheral blood mononuclear cells (PBMCs) from *H. pylori* negative donors and infected them with *H. pylori* for 24 h. The supernatant obtained from this mixture of immune cells was collected and used to incubate the GC cell lines. We found strong activation of STAT1 and upregulation of PD-L1 expression in cells incubated with supernatants from *H. pylori*-infected PBMCs (Figure 1g), indicating that activation of STAT1 and PD-L1 expression in the gastric epithelium depends on cytokines released by immune cells after encounter with *H. pylori*.

### 2.2. H. pylori-Induced Activation of STAT1 and PD-L1 Expression Correlate with Immune Infiltration and Cell Proliferation in Gastric Lesions

To further investigate the correlation of STAT1 activation and PD-L1 upregulation with immune cell infiltration, we employed Rnf43^H292R/H295R^ mice, which were previously described to develop precancerous lesions in the stomach characterized by high levels of IFN-γ upon *H. pylori* infection [19].

**Figure 2:**
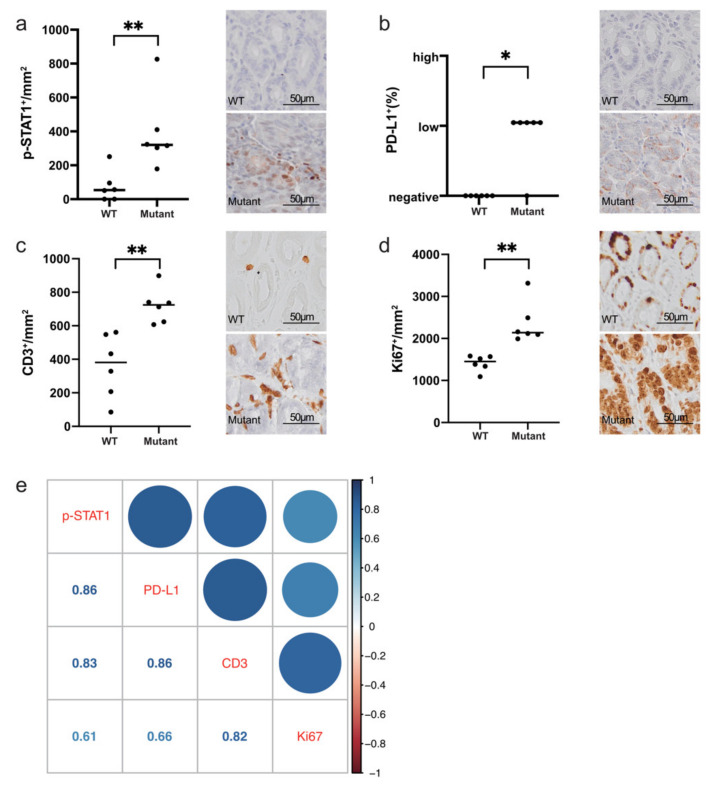
Immunohistochemistry staining of p-STAT1, PD-L1, immune infiltration, and proliferation in *H. pylori*-induced gastric lesions of Rnf43 mutant mice (20× magnification). (**a**) p-STAT1, (**b**) PD-L1, (**c**) CD3, (**d**) Ki67 in the stomachs of wild-type (*n* = 6) and Rnf43 mutant mice (*n* = 6) after 6-month *H. pylori* infection. (**e**) Correlation of gene expressions with immune infiltration and proliferation. Color represents spearman’s correlation coefficient. Wilcoxon rank-sum test, * *p* < 0.05, ** *p* < 0.01. PD-L1 staining evaluation: <1%, negative; 1–49% low; ≥50% high. Mutant, Rnf43^H292R/H295R^ mutant mice; WT, wild-type mice.

In *H. pylori*-induced gastric lesions of Rnf43^H292R/H295R^ mice, we observed increased activation of STAT1 and upregulation of PD-L1 expression compared to infected wild-type mice (Figure 2a,b). Rnf43 mutant mice also presented higher lymphocytic infiltration, as detected by immunohistochemical staining of CD3+ cells (Figure 2c). In addition, hypertrophic glands of Rnf43^H292R/H295R^ mice were highly proliferative, showing high numbers of Ki67 positive cells (Figure 2d). Notably, phosphorylation of STAT1 and PD-L1 expression correlated with lymphocytic infiltration and cell proliferation (Figure 2e).

### 2.3. STAT1 and PD-L1 Expression Correlate with Immune Infiltration in Humans and Can Be Used as Biomarkers for GC Prognosis

**Figure 3:**
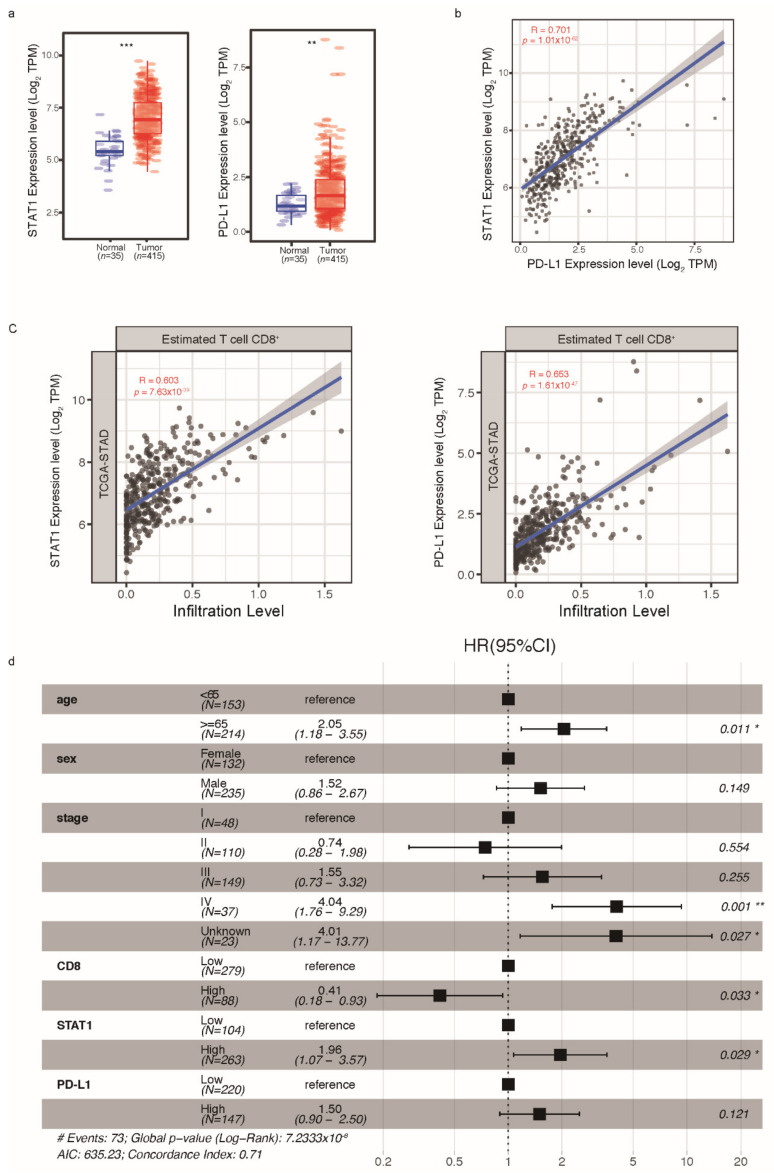
Transcriptome analysis of STAT1 and PD-L1 based on the TCGA-STAD database. (**a**) Expression of STAT1 and PD-L1 in tumor tissues (*n* = 415) and normal tissues (*n* = 35). (**b**) Correlation of STAT1 and PD-L1 expression in tumor tissues. (**c**) Correlations of STAT1 and PD-L1 with immune infiltration levels. (**d**) Multivariable Cox regression analysis of GC. Wilcoxon rank-sum test, * *p* < 0.05, ** *p* < 0.01, *** *p* < 0.001. CI, confidence interval; GC, gastric cancer; HR, hazard ratio; TCGA-STAD, The Cancer Genome Atlas-Stomach Adenocarcinoma.

Due to limited GC samples in our proteomic cohort to establish associations (Figure 1a), we referred to The Cancer Genome Atlas Stomach Adenocarcinoma (TCGA-STAD) database to analyze the expression of STAT1 and PD-L1 and their correlation with immune infiltration based on mRNA level [20]. STAT1 and PD-L1 were both significantly upregulated in GC tissues compared with normal tissues (Figure 3a), and the expression of STAT1 and PD-L1 in tumor tissues were highly correlated (Figure 3b). Using a deconvolution method [21], we estimated the immune infiltration of tumor tissues based on mRNA data. STAT1 and PD-L1 were both highly correlated with CD8+ T-cell immune infiltration (Figure 3c). When analyzing tumor prognosis, CD8+ T cell immune infiltration and STAT1 were identified to be independently associated with prognosis (HR [95%CI] = 0.41 [0.18–0.93] for CD8 + T cell immune infiltration, and HR [95%CI] = 1.96 [1.07–3.57] for STAT1 expression). PD-L1 was also correlated with poor survival (HR [95%CI] = 1.50 [0.90–2.50]) (Figure 3d).

**Figure 4:**
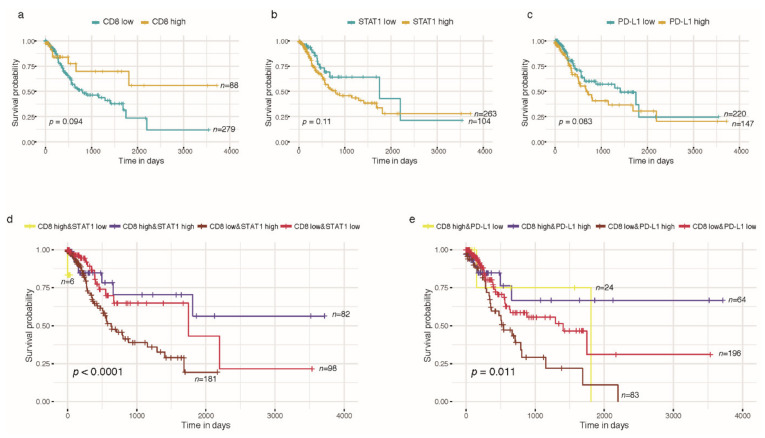
Kaplan-Meier curves of CD8+ T cell immune infiltration, STAT1 and PD-L1 in TCGA-STAD prognosis analysis. (**a**) CD8+ T cell immune infiltration; (**b**) STAT1; (**c**) PD-L1; (**d**) CD8+ T cell immune infiltration combined STAT1; (**e**) CD8+ T cell immune infiltration combined PD-L1. Log rank test for *p*-value.

CD8+ T cell immune infiltration displayed better differentiation for GC prognosis compared to STAT1 and PD-L1 alone (Figure 4a–c). Patients with high CD8+ T cell immune infiltration showed better survival, while those with low CD8+ T cell immune infiltration accompanied with high STAT1 or PD-L1 had worse survival (Figure 4d,e).

## 3. Discussion

Gastric cancer is the fifth most common cancer worldwide and the fourth leading cause of cancer-related deaths [22]. GC evolves over decades from superficial gastritis to adenocarcinoma, and the majority of patients are diagnosed at an advanced stage with a poor prognosis. *H. pylori* infection induces immune cell infiltration in the human stomach, and the consecutive dysregulation of cytokine receptor signaling is a key event not only regulating the immune response to the infection but also orchestrating changes related to chronic inflammation, development of GC and progression, and immune escape of gastric tumors. In the current study, we showed that STAT1 is activated as an early response to *H. pylori* infection. STAT1 is generally considered to function as a tumor suppressor, but there is growing evidence showing that STAT1 can also act as a tumor promotor [23,24,25]. Several studies suggested this tumor-promoting function to be related to STAT1-mediated regulation of PD-L1 expression [13,14,26,27]. In agreement, our results support a tumor-promoting effect of STAT1 expression and activation since it is upregulated at early stages of *H. pylori*-induced inflammation and increases along the progression of gastric lesions to GC. More importantly, STAT1 expression is associated with poor survival, supporting a detrimental effect of STAT1 signaling during gastric tumorigenesis. One possible mechanism leading to this effect could be the upregulation of target genes, such as PD-L1. Indeed, we observed STAT1 activation to be accompanied by the upregulation of PD-L1 in gastric epithelial cells suggesting that PD-L1 might be a target gene of STAT1 upon *H. pylori* infection. Importantly, PD-L1 also correlated with poor prognosis, and both STAT1 and PD-L1 expression were also highly correlated with immune cell infiltration. *H. pylori*-induced immune cell infiltration is well recognized as an important driver of inflammation-associated gastric tumorigenesis, while tumor-infiltrating lymphocytes have been studied for their roles as prognostic markers and potential therapeutic targets in GC. In particular, CD8+ cytotoxic T cells, which are the main effector cells directly killing transformed cells [28], were demonstrated to be associated with better survival of patients with GC [29,30]. Our results are in line with these previous studies and further correlate STAT1 and PD-L1 expression with CD8+ T cell infiltration. In light of our results, we hypothesize that upregulation of PD-L1 expression via STAT1 activation in gastric epithelial transformed cells suppresses anti-tumor immune responses of CD8+ cytotoxic T cells. This facilitates tumor immune escape and consequently enables tumor progression and growth.

Immunotherapy, especially immune checkpoint inhibitors, provides a new opportunity for GC treatment. Our findings showing that STAT1 is independently associated with poor prognosis suggest the analysis of JAK-STAT1 signaling in GC patients to better assess prognosis. Moreover, the use of inhibitors or antagonists of this pathway should be considered to enhance the success of PD1/PD-L1 blockade therapy.

## 4. Materials and Methods

### 4.1. Proteomic Profiling of Precancerous Gastric Lesions and GC

We explored STAT1 expression in a gastric tissue cohort that we previously used for proteomic analysis [18]. Briefly, liquid chromatography tandem mass spectrometry was used for tissue proteomic profiling of 169 subjects including 33 SGs, 19 CAGs, 56 IMs, 3 LGINs and 58 GCs. A label-free intensity-based absolute quantification (iBAQ) approach was used to quantify protein abundance. The iBAQ values were then converted to intensity-based fraction of total (iFOT), calculated as the iBAQ of each protein divided by the sum of iBAQs of all proteins in the sample and multiplied by 10^5^ to ease the visualization of low abundant proteins. 

### 4.2. Cell Culture and H. pylori Infection

Gastric cancer cell lines NUGC4 (JCRB0834) and NCI-N87 (ATCC^®^CRL-5822) were cultured in Dulbecco’s modified Eagle medium (Invitrogen, Carlsbad, CA, USA) containing 10% fetal calf serum (FCS) and 1% penicillin/streptomycin, and maintained at 37 °C in a humidified atmosphere (5% CO_2_). Cells were tested routinely for mycoplasma contamination. *H. pylori* strain G27 [31] was cultured on Wilkins–Chalgren (WC) Dent agar plates (OXOID, Hampshire, UK) in a microaerophilic atmosphere at 37 °C and 10% CO_2_. Cells were infected at a multiplicity of infection (MOI) of 10 (OD_600_ 1 = 2 × 10^8^ bacteria/mL) for 24 h and lysed in sodium dodecyl sulfate (SDS) buffer for protein expression analysis. IFN-γ (10 ng/mL) cells stimulated for 24 h were used as positive control.

Human peripheral blood mononuclear cells were isolated from *H. pylori*-negative healthy donors, after informed consent, by density gradient centrifugation with Pancoll (PAN-Biotech, Aidenbach, Germany). Cells were cultured with RPMI-1640 containing 10%FCS at 37 °C in a humidified atmosphere (5% CO_2_) and infected with the *H. pylori* G27 strain at MOI 10 for 24 h. The supernatant was collected and co-cultured with gastric cancer cell lines (NUGC4 and NCI-N87) for 24 h. After co-culturing, gastric cancer cells were lysed in SDS buffer for protein expression analysis.

### 4.3. Western Blot

To check for protein expression, equal volumes of protein lysate were loaded on an 8% sodium dodecyl sulfate–polyacrylamide gel, and electrophoresis was performed. Separated proteins were transferred onto a nitrocellulose membrane (Amersham Protran 0.45, GE Healthcare, Chicago, IL, USA). The membrane was blocked with 5% non-fat milk in TBS-T buffer (Tris-buffered saline supplemented with 0.1% Tween 20) at room temperature for one hour. The blocked membrane was probed with primary antibodies targeting p-STAT1 (#9167, Cell Signaling Technology, Danvers, MA, USA, 1:1000) and PD-L1 (#13684, Cell Signaling Technology, Danvers, MA, USA, 1:1000) at 4 °C overnight. GAPDH (#2118, Cell Signaling Technology, Danvers, MA, USA, 1:1000) was used as a protein loading control. After washing, the membrane was incubated with secondary HRP-conjugated anti-rabbit antibody (Promega, Madison, WI, USA). Proteins were detected by applying an ECL Western blotting detection reagent (Thermo Fisher Scientific, Waltham, MA, USA).

### 4.4. Immunohistochemistry

Human gastric biopsy samples from paraffin-fixed, paraffin-embedded (FFPE) blocks were obtained from the tissue bank of the Institute of Pathology, Klinikum Bayreuth in Germany, after the approval of the local ethics committee (155_20B). FFPE gastric samples from mice [19,32] previously described were used in the present study. Briefly, Rnf43 mutant mice were generated by introducing two point mutations in the RING domain of Rnf43 through homology-directed repair. Six- to eight-week-old mice were infected twice with 2 × 10^8^ *H. pylori* strain PMSS1 diluted in 200 μL brain-heart infusion (BHI) containing 20% FCS by oral gavage and sacrificed after 6 months. Stomachs were dissected, and tissues were fixed in 4% formaldehyde, and paraffin embedded. Human and mice gastric tissue samples from wild-type (WT) and Rnf43 mutant mice were incubated with specific antibodies (Table 1) overnight after antigen retrieval in a pressure cooker using 10 mM sodium citrate (pH6) or 1 mM EDTA (pH8) (p-STAT1 in humans and mice, and PD-L1 in humans). After incubation with HRP-conjugated secondary antibodies, sections were developed using SignalStain DAB substrate (Cell Signaling Technology, Danvers, MA, USA), and counterstained with hematoxylin (Morphisto, Frankfurt, Germany). The slides were scanned and analyzed using an Olympus Virtual Slide Imaging System (Olympus, Tokyo, Japan). Five random high-power field areas per sample were quantified in a blind manner. Positive staining cells were counted dividing by area and mean values of five random areas for each slide were used for statistical analysis.

### 4.5. Statistical Analyses

Statistical analyses were performed using R (version 3.6.0) software. Differential gene or protein expression analysis was tested by Wilcoxon rank-sum test, and with two-sides, *p* < 0.05 was considered statistically significant. The correlation of gene or protein expression was evaluated using Spearman’s correlation coefficients. The immune infiltration level of TCGA gastric tumors was estimated by a deconvolution method previously published [21]. Probabilities of overall survival were estimated using the Kaplan–Meier (KM) method and compared using the log-rank test or the Cox proportional hazards regression model. The Youden index was used as the cut-off point to stratify patients with different prognoses.

## 5. Conclusions

*H. pylori* infection induces PD-L1 expression in the gastric epithelium and during GC development in a STAT1 and immune cell-dependent manner. PD-L1 may allow transformed epithelial cells to progress to GC and later determine GC prognosis.

## Figures and Tables

**Table 1:** Antibodies used for immunohistochemistry.

Target	Clone	Company
p-STAT1	58D6	Cell Signaling Technology
PD-L1 (human)	E1L3N	Cell Signaling Technology
PD-L1 (mouse)	D5V3B	Cell Signaling Technology
CD3	SP7	Thermo Fisher Scientific
Ki67	D2H10	Cell Signaling Technology

## Data Availability

Not applicable.
